# Procalcitonin, mid-regional proadrenomedullin and C-reactive protein in predicting treatment outcome in community-acquired febrile urinary tract infection

**DOI:** 10.1186/s12879-019-3789-6

**Published:** 2019-02-14

**Authors:** Janneke Evelyne Stalenhoef, Cees van Nieuwkoop, Darius Cameron Wilson, Willize Elizabeth van der Starre, Tanny J. K. van der Reijden, Nathalie Manon Delfos, Eliane Madeleine Sophie Leyten, Ted Koster, Hans Christiaan Ablij, Johannes (Jan) Willem van ‘t Wout, Jaap Tamino van Dissel

**Affiliations:** 1 Department of Infectious Diseases, Leiden University Medical Center, PO Box 9600, 2300 RC Leiden, the Netherlands; 2 Department of Internal Medicine, Haga Hospital, The Hague, The Netherlands; 3 Thermo Fisher Scientific, Hennigsdorf, Germany; 4 Department of Internal Medicine, Alrijne Hospital, Leiderdorp, The Netherlands; 5 Department of Internal Medicine, MCH, The Hague, The Netherlands; 6 Department of Internal Medicine, Groene Hart Hospital, Gouda, The Netherlands; 7 Department of Internal Medicine, Alrijne Hospital, Leiden, The Netherlands

**Keywords:** Urinary tract infections, Pyelonephritis, Biomarkers, Treatment duration, Antibiotic therapy, Antibiotic stewardship

## Abstract

**Background:**

A reduction in duration of antibiotic therapy is crucial in minimizing the development of antimicrobial resistance, drug-related side effects and health care costs. The minimal effective duration of antimicrobial therapy for febrile urinary tract infections (fUTI) remains a topic of uncertainty, especially in male patients, those of older age or with comorbidities. Biomarkers have the potential to objectively identify the optimal moment for cessation of therapy.

**Methods:**

A secondary analysis of a randomized placebo-controlled trial among 35 primary care centers and 7 emergency departments of regional hospitals in the Netherlands. Women and men aged ≥18 years with a diagnosis of fUTI were randomly assigned to receive antibiotic treatment for 7 or 14 days. Patients indicated to receive antimicrobial treatment for more than 14 days were excluded from randomization. The biomarkers procalcitonin (PCT), mid-regional proadrenomedullin (MR-proADM), and C-reactive protein (CRP) were compared in their ability to predict clinical cure or failure through the 10–18 day post-treatment visit.

**Results:**

Biomarker concentrations were measured in 249 patients, with a clinical cure rate of 94% in the 165 randomized and 88% in the 84 non-randomized patients. PCT, MR-proADM and CRP concentrations did not differ between patients with clinical cure and treatment failure, and did not predict treatment outcome, irrespective of 7 or 14 day treatment duration (ROC_AUC_ 0.521; 0.515; 0.512, respectively). PCT concentrations at presentation were positively correlated with bacteraemia (τ = 0.33, *p* < 0.001) and presence of shaking chills (τ = 0.25, *p* < 0.001), and MR-proADM levels with length of hospital stay (τ = 0.40, *p* < 0.001), bacteraemia (τ = 0.33, *p* < 0.001), initial intravenous treatment (τ = 0.22, *p* < 0.001) and time to defervescence (τ = 0.21, *p* < 0.001). CRP did not display any correlation to relevant clinical parameters.

**Conclusions:**

Although the biomarkers PCT and MR-proADM were correlated to clinical parameters indicating disease severity, they did not predict treatment outcome in patients with community acquired febrile urinary tract infection who were treated for either 7 or 14 days. CRP had no added value in the management of patients with fUTI.

**Trial registration:**

The study was registered at ClinicalTrials.gov [NCT00809913; December 16, 2008] and trialregister.nl [NTR1583; December 19, 2008].

## Background

Febrile urinary tract infections (UTI), including acute pyelonephritis and prostatitis, are relatively common in adults, but data on the optimal duration of treatment are limited, especially within men, the elderly and patients with comorbidities. Emerging bacterial resistance calls for more efficient efforts to shorten the duration of antibiotic treatment.

Our previous findings in the FUTIRST trial have shown that patients with community-acquired febrile urinary tract infections, such as women and elderly patients with severe comorbidities, can be safely and efficaciously treated with oral ciprofloxacin for 7 days, irrespective of disease severity upon presentation [1]. In men, however, a short course of therapy can lead to significantly more clinical failures compared to a 14-day course of oral ciprofloxacin.

Previous studies have shown that procalcitonin (PCT) can provide useful guidance for antimicrobial treatment in patients with respiratory tract infections and sepsis [2–4]. However, little is known concerning PCT guided-therapy in patients with urinary tract infections. An earlier subgroup analysis of a randomized trial performed in the intensive care unit (ICU) has shown that the duration of antibiotic treatment was significantly shorter for 24 UTI-patients receiving procalcitonin-guided treatment compared to 18 UTI control-group patients [4]. However further investigations are scarce. Other biomarkers may also be of interest, such as mid-regional proadrenomedullin (MR-proADM), which has been shown to be increased in the early stages of progression towards multiple organ failure [5], and is of value in predicting a complicated course of treatment and the requirement for ICU admission [6–8].

This secondary analysis of the earlier FUTIRST trial [1] therefore hypothesized that procalcitonin measurement on days 0 and 3 could more accurately identify patients at risk of treatment failure and in need a prolonged course of antibiotics compared to either MR-proADM or C-reactive protein.

## Methods

### Design and study population

This was a secondary analysis of a randomized, placebo-controlled trial involving patients presenting with febrile urinary tract infection (fUTI) at the emergency departments (ED) of 7 hospitals and 32 primary health care centres in the Netherlands, between November 2008 and May 2013, as described previously [1, 9]. This trial enrolled consecutive adult patients with a presumptive diagnosis of community-acquired fUTI established by a primary care or emergency physician, who met the following criteria: fever of ≥38.2 °C and/or a history of feeling feverish with shivering or rigors in the past 24 h, one or more symptoms suggestive of UTI (i.e. dysuria, frequency, urgency, perineal or suprapubic pain, costovertebral tenderness, or flank pain), and positive urine nitrate test and/or pyuria (positive leucocyte esterase test or > 5 leucocytes per high-power field in a centrifuged sediment). Exclusion criteria for study entry were as follows: known allergy to fluoroquinolones, pregnancy or lactation, polycystic kidney disease, permanent renal replacement therapy and kidney transplantation. Patients enrolled with fUTI but not randomized to trial medication remained in the observational part of the study to assess outcome [1]. In the current study, all patients participating in both observational and interventional part of the trial were included, except those without a baseline blood sample available for biomarker analysis, without a 10–18 days post-treatment visit, and patients who needed prolonged treatment because of chronic bacterial prostatitis.

The study protocol was approved by the local Ethical Committee, and written informed consent was obtained from all participants. The trial was registered at ClinicalTrials.gov (NCT00809913; December 16, 2008) and trialregister.nl (NTR1583; December 19, 2008).

### Procedures

Within 24–48 h of notification, qualified research nurses collected clinical data and laboratory values by standardized questionnaires. The decision whether to treat as outpatient or inpatient was left to the discretion of the attending physician. Outpatients started with the first week of open label ciprofloxacin 500 mg twice daily. In hospitalized patients, the treating physician could administer empirical intravenous antibiotics at the start of treatment according to local policy (in all participating centres: a ß-lactam antibiotic ± aminoglycoside). These patients were switched to open label ciprofloxacin as soon as was deemed possible. Randomization between the second week ciprofloxacin and placebo twice daily was initiated once the results of the urine culture became available on the third or fourth day after inclusion. In patients who could not be randomized (e.g. due to ciprofloxacin resistance), the choice of antibiotic agent and treatment duration was left at the discretion of the treating physician [1].

All patients were contacted in person on day 3 (3–4 days after start of treatment) and day 30 (10–18 days post-treatment) after enrolment, and by phone on day 90 (70–84 days post-treatment) to assess clinical outcome. EDTA plasma samples were collected, centrifuged and stored at − 80 °C within 2 h of patient enrolment. MR-proADM and PCT were batch-measured in a blinded fashion by TRACE technology (Time Resolved Amplified Cryptate Emission) using a new sandwich immunoassay (Kryptor Compact Plus Analyzer, BRAHMS, Hennigsdorf, Germany), with a limit of detection of 0.05 nmol/L and 0.02 ng/L, respectively. Further details on randomization, trial medication and study procedures have been published previously [1, 9].

### Outcome measure

The biomarkers PCT and MR-proADM were evaluated for their ability to predict the clinical cure. Clinical cure was assessed on the day 30 visit (10–18 days post-treatment), and was defined as survival with absence of fever and a resolution of UTI symptoms (either absence of symptoms or at least 2 points improvement on a 0–5 point severity score scale), without additional antimicrobial therapy (for relapse of UTI) [1]. Patients who did not meet the criteria of clinical cure at day 30, were considered to have clinical treatment failure. Long term clinical cure was assessed at the 90-day post-treatment interview.

### Statistical analysis

Descriptive statistics are expressed as frequencies (percentage), means with standard deviation (SD) or as medians with interquartile range (IQR), as appropriate. Univariate analysis was performed using ANOVA, student’s t-test or Mann-Whitney U test where appropriate for continuous variables and Chi-square test for categorical variables. Non-parametric tests were used to analyse biomarkers.

Analyses were performed in the total patient population and in 2 subgroups based on treatment duration. Kendall’s rank tau-b was used to investigate correlations between biomarker levels and clinical parameters. Finally, area under the receiver operating characteristic (AUROC) curves with 95% confidence intervals (CI) were calculated to assess the prognostic ability of PCT and MR-proADM.

A *p*-value of < 0.05 was considered to indicate statistical significance. SPSS software (SPSS Inc. Chicago, version 23.0) was used for statistical analysis.

## Results

**Fig. 1:**
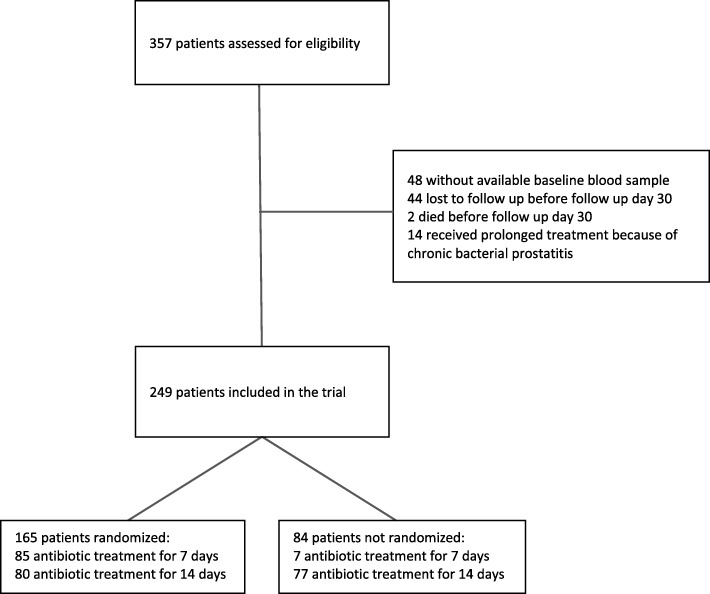
Flow of patients

A total of 249 patients with a presumptive diagnosis of fUTI were analysed (details provided in Fig. 1). Patient characteristics in terms of urologic history, comorbidities and presenting symptoms are outlined in Table 1. Of these, 165 (66%) were randomly assigned to receive antimicrobial treatment for either 7 (*N* = 85) or 14 (*N* = 80) days; in the remaining patients the treatment duration was left at the discretion of the treating physician. The majority of patients (*N* = 175; 70%) were included at the ED, and 73 (29%) patients undergoing existing antimicrobial treatment prior to presentation. Patients had an average age of 60 (45–73) years, with females comprising the majority of enrolled patients (*N* = 148; 59%).

**Table 1:** Baseline characteristics of 249 patients with febrile urinary tract infection

	All patients (*n* = 249)
Age (years)	60 (45–73)
Male sex	101 (41)
Urologic history	
Indwelling urinary catheter	7 (3)
Urinary tract disorder^a^	75 (30)
Recurrent UTI^b^	57 (23)
Comorbidity	
Diabetes mellitus	33 (13)
Malignancy	17 (7)
Heart failure	21 (8)
Cerebrovascular disease	13 (5)
Chronic renal insufficiency	9 (4)
COPD	27 (11)
Immunocompromised	15 (6)
Presentation	
At emergency department	175 (70)
Antibiotic pretreatment	73 (29)
Fever duration, hours	36 (19–72)
Dysuria	188/243 (77)
Flank pain	159/245 (65)
Suprapubic pain	127/242 (52)
Perineal pain	11/241 (5)
Systolic BP, mmHg	130 (116–146)
Diastolic BP, mmHg	74 (64–84)
Heart rate, beats/minute	94 (80–107)
Cultures	
Positive urine culture	171 (69)
Escherichia coli	143/171 (84)
Positive blood culture	45/240 (19)
Positive urine and/or blood culture	183 (73)

Data presented as number (%) or median (IQR).

*BP* blood pressure, *AB* antibiotics, *TMP/SMX* trimethoprim-sulfamethoxazole.

^a^any functional or anatomical abnormality of urinary tract except urinary catheter

^b^≥3 UTIs in past 12 months or ≥ 2 UTIs in past 6 months

The clinical cure rate was high (*N* = 229; 92%) and did not differ significantly between randomized and non-randomized patients (94% vs. 88%, respectively). A total of 20 patients did not reach the endpoint of clinical cure, due to persistence or recurrence of UTI symptoms (N = 8), or to the use of additional antibiotics for relapse of UTI (*N* = 12), assessed on the day 30 visit. No significant differences were seen in the clinical characteristics between patients with treatment success or failure. In addition, mean treatment duration was similar (11 days) in both patients with clinical cure and clinical failure (Table 2).

**Table 2:** Characteristics of patients with clinical cure and clinical failure

	Clinical cure (*n* = 229)	Clinical failure (*n* = 20)	*P* value
Age (years)	60 (45–73)	56 (46–71)	0.412
Male sex	94 (41)	7 (35)	0.597
Urologic history			
Indwelling urinary catheter	7 (3)	0	0.428
Urinary tract disorder^a^	71 (31)	4 (20)	0.304
Recurrent UTI^b^	50 (22)	7 (35)	0.191
Comorbidity			
Diabetes mellitus	32 (14)	1 (5)	0.256
Malignancy	16 (7)	1 (5)	0.735
Heart failure	21 (9)	0	0.157
Cerebrovascular disease	12 (5)	1 (5)	0.963
Chronic renal insufficiency	8 (3)	1 (5)	0.729
COPD	27 (11)	2 (10)	0.899
Immunocompromised	14 (6)	1 (5)	0.841
Presentation			
At emergency department	160 (70)	15 (75)	0.630
Antibiotic pre-treatment	71 (31)	2 (10)	0.048
Fever duration, hours	36 (18–72)	48 (24–120)	0.279
Dysuria	175/224 (78)	13/19 (68)	0.332
Flank pain	142/225 (63)	17 (85)	0.049
Suprapubic pain	116/222 (52)	11 (55)	0.814
Perineal pain	11/222 (5)	0/19 (0)	0.321
Systolic BP, mmHg	130 (116–146)	130 (115–150)	0.753
Diastolic BP, mmHg	74 (63–85)	72 (68–83)	0.585
Heart rate, beats/minute	93 (80–107)	96 (78–110)	0.695
Cultures			
Positive urine culture	158 (69)	13 (65)	0.712
Escherichia coli	133/158 (84)	10/13 (77)	0.497
Positive blood culture	42/220 (19)	3 (15)	0.654
Positive urine and/or blood culture	169 (74)	14 (70)	0.712
Treatment			
Short antimicrobial treatment (7 days)	82 (36)	10 (50)	0.207
Days of AB, mean (SD)	11 (3.3)	11 (3.5)	0.252
Ciprofloxacin	208 (91)	18 (90)	0.902
Amoxicillin (± clavulanic acid)	11 (5)	2 (10)	0.316
TMP/SMX	5 (2)	0	0.504
Other^c^	5 (2)	0	0.504
Initial intravenous dose(s) of AB	135 (59)	12 (60)	0.927
Randomized	155 (68)	10 (50)	0.109
Outpatient treatment	89 (39)	7 (35)	0.733

Data presented as number (%) or median (IQR)

*BP* blood pressure, *AB* antibiotics, *TMP/SMX* trimethoprim-sulfamethoxazole

^a^any functional or anatomical abnormality of urinary tract except urinary catheter

^b^≥3 UTIs in past 12 months or ≥ 2 UTIs in past 6 months

^c^cefuroxime iv n = 2, meropenem iv n = 1, moxifloxacin n = 1, flucloxacillin *n* = 1

Median biomarker concentrations across the total patient population were as follows: PCT: 0.40 [0.12–1.54] ug/mL; MR-proADM: 0.89 [0.63–1.28] nmol/L; and CRP: 118 (52–205) mg/L.

### Prediction of treatment outcome

#### Total patient population

**Fig. 2:**
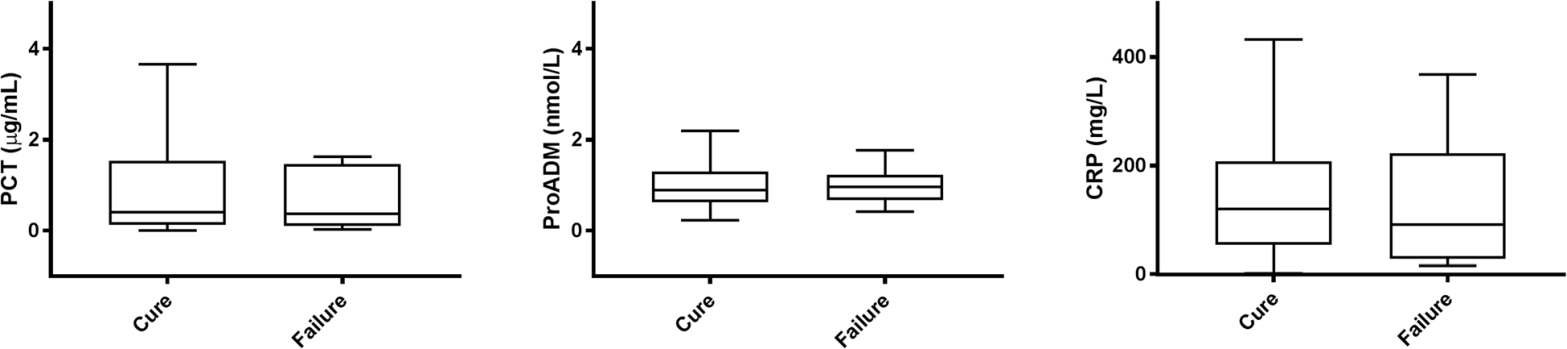
Levels of PCT, MR-proADM and CRP measured at presentation in patients with clinical cure and failure

Concentrations of PCT, MR-proADM and CRP, measured at presentation, did not differ between patients with clinical cure or treatment failure (Fig. 2 and Table 3). As shown in Table 3, biomarker levels also did not predict treatment failure when measured after 3 days of treatment. We also assessed cut-offs for PCT as previously used in other studies, such as 0.25 μg/mL [10, 11] and a decrease of PCT concentration by 80% [4].

**Table 3:** Biomarkers in patients with clinical cure and clinical failure

	Clinical cure	Clinical failure	P value
All patients (*n* = 249)	n = 229	n = 20	
At presentation			
PCT	0.40 (0.13–1.54)	0.36 (0.10–1.46)	0.749
PCT > 0.25	136 (59%)	12 (60%)	0.957
CRP	120 (53–211)	90 (27–223)	0.851
proADM	0.89 (0.62–1.30)	0.86 (0.67–1.20)	0.948
Day 3			
PCT	0.18 (0.07–0.87)	0.12 (0.04–0.69)	0.667
PCT ≤ 0.25	122 (60%)	12 (67%)	0.568
PCT ≤ 0.25 or PCT decline ≥80%	144 (71%)	14 (78%)	0.519
proADM	0.66 (0.50–0.91)	0.63 (0.51–0.78)	0.667
Short treatment (*n* = 92)	*n* = 82	*n* = 10	
At presentation			
PCT	0.34 (0.10–1.68)	0.36 (0.11–1.79)	0.980
PCT > 0.25	47 (57%)	6 (60%)	0.871
CRP	123 (53–194)	77 (15–142)	0.231
proADM	0.76 (0.56–1.04)	0.85 (65–1.25)	0.360
Day 3			
PCT	0.12 (0.06–0.42)	0.13 (0.09–0.63)	0.794
PCT ≤ 0.25	51 (66%)	7 (78%)	0.484
PCT ≤ 0.25 or PCT decline ≥80%	62 (80%)	8 (89%)	0.542
proADM	0.56 (0.47–0.76)	0.59 (0.53–0.74)	0.679
Long treatment (*n* = 157)	*n* = 147	n = 10	
At presentation			
PCT	0.48 (0.15–1.49)	0.48 (0.09–3.13)	0.826
PCT > 0.25	89 (60%)	6 (60%)	0.973
CRP	119 (52–224)	181 (37–263)	0.531
proADM	0.98 (0.67–1.44)	0.86 (0.62–1.21)	0.495
Day 3			
PCT	0.22 (0.08–1.08)	0.08 (0.04–0.72)	0.358
PCT ≤ 0.25	71 (56%)	5 (56%)	0.984
PCT ≤ 0.25 or PCT decline ≥80%	82 (65%)	6 (67%)	0.899
proADM	0.74 (0.53–1.03)	0.74 (0.47–1.02)	0.609

Data presented as median (IQR) or number (%)

CRP at presentation missing: *n* = 41 in short treatment and n = 41 in long treatment

**Fig. 3:**
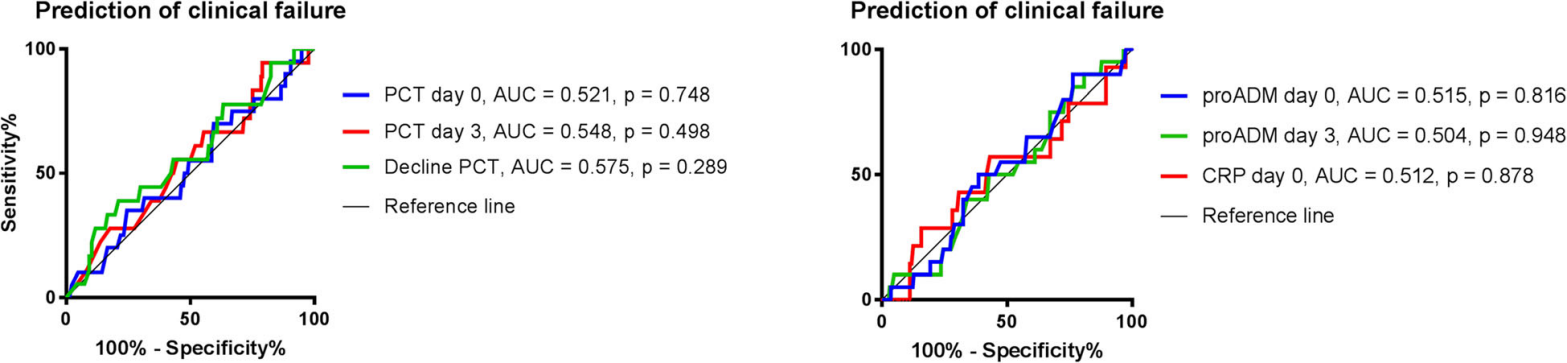
Biomarker accuracy in the prediction of treatment outcome

ROC analyses were further performed to define the prognostic accuracy of the different biomarkers for predicting treatment outcome. Based on the calculated AUCs, none of the biomarkers had any predictive value for treatment success (Fig. 3). Findings were similar in a selection of patients with culture proven UTI (*N* = 183); and regarding the long term clinical cure in all patients (data not shown).

#### Patients treated for 7 days

We assessed the group of patients who received 7 days of antimicrobial treatment separately, because treatment success was lower as compared to those treated for 14 days (89% versus 94%, respectively, *p* = 0.21). Again, no difference was seen in levels of PCT, MR-proADM and CRP between patients with clinical cure or treatment failure (Table 3).

### Correlation between biomarkers and clinical parameters

#### Procalcitonin

PCT concentrations at presentation were positively correlated with bacteraemia (τ = 0.33, *p* < 0.001) and presence of shaking chills (τ = 0.25, *p* < 0.001). Although significant, correlations with initial intravenous treatment, length of hospital admission, time to defervescence, ICU admission, temperature, heart rate and confusion at presentation were weak (τ < 0.20). Furthermore, PCT increased slightly with age (τ = 0.12, *p* < 0.01) and was not correlated to comorbidity. Median PCT concentrations measured after 3 days of treatment showed a similar trend, whereas the decrease of PCT concentration was not correlated to clinical parameters.

#### Mid-regional proadrenomedullin

MR-proADM levels at presentation were positively correlated with length of hospital stay (τ = 0.40, *p* < 0.001), bacteraemia (τ = 0.33, p < 0.001), initial intravenous treatment (τ = 0.22, *p* < 0.001) and time to defervescence (τ = 0.21, *p* < 0.001). Weak, though significant correlation was seen with temperature, need for ICU admission, confusional state at presentation and the presence of shaking chills (τ < 0.20). Compared to PCT, MR-proADM showed a stronger correlation with age (τ = 0.49, *p* < 0.001), and was weakly correlated to pre-existing comorbidity (diabetes mellitus, urinary tract disorder and chronic renal insufficiency). Again, after 3 days of treatment, correlations were similar. Decreasing MR-proADM concentrations between presentation and day 3 were correlated to bacteraemia (τ = 0.22, *p* < 0.001) and the presence of shaking chills at presentation (τ = 0.22, *p* < 0.001).

#### C-reactive protein

CRP was only weakly positively correlated to time to defervescence (τ = 0.13, *p* = 0.02) and weakly negatively correlated to temperature (τ = − 0.11, *p* = 0.04) and mean arterial pressure (τ = − 0.13 *p* = 0.02). No correlations were found between CRP and other parameters, such as age or comorbidity.

## Discussion

In the current study we assessed the ability of the biomarkers PCT, MR-proADM and CRP in predicting treatment failure in a randomized trial of antibiotic treatment duration (i.e. 7 versus 14 days) in patients with febrile urinary tract infection. We hypothesized that the biomarker signature, as an objective laboratory proxy of physical status in fUTI, would help the physician in guiding the length of antibiotic therapy. In our study, however, neither PCT nor MR-proADM or CRP were distinctive in identifying upfront those patients at risk for a treatment failure, i.e. who might benefit from a more prolonged antibiotic treatment.

Overall treatment success was high: 94% of the randomized 165 and 88% of 84 non-randomized patients who all were given an identical follow-up reached the endpoint of clinical cure. Of the biomarkers, PCT and MR-proADM were significantly correlated to clinical parameters such as fever, blood pressure and subjective complaints that represent the acute febrile illness of invasive urinary tract infection. Also, the biomarker signature of PCT and MR-proADM correlated with severity of disease, such as presence of bacteraemia and need for initial administration of antibiotics intravenously rather than orally. Finally, the course of biomarkers over the first three days correlated with signs of clinical recovery, such as time to defervescence, intensity of subjective complaints and length of hospital stay. As opposed to PCT and MR-proADM, the currently popular biomarker CRP did not display any correlation to relevant clinical parameters like course of fever or bacteraemia.

The biomarkers we chose are in current use as predictors of morbidity and mortality in a variety of conditions. Procalcitonin is a precursor hormone of calcitonin and is upregulated by cytokines released in response to bacterial infection [4]. MR-proADM has been detected in a variety of tissues, including heart, vessels and the kidneys and has both immune modulating and vasodilating properties [2]. Levels of MR-proADM are elevated in sepsis, contributing to hypotension in these patients. Thus, procalcitonin has shown to be a marker of bacteraemia in patients with febrile UTI [12–14], whereas mid-regional proadrenomedullin (MR-proADM) is a predictor of a complicated course of disease, the need for ICU admission, and mortality [6, 7].

PCT-based algorithms have been developed to aid decisions on the initiation and/or discontinuation of antibiotics in patients with acute respiratory tract infections and in critically ill patients admitted to the intensive care ward [15]. In these specific patient groups, the use of PCT has been shown to be a powerful tool in antibiotic stewardship [16]. Although in the intensive care studies patients with infections originating from the urinary tract were included, numbers were small (e.g. 7% of patients in the PRORATA trial, 3% of patients in the SAPS trial) and these results cannot directly be extrapolated to patients with community-acquired urinary tract infections [4, 17].

Elevation of these biomarkers is a reflection of the systemic inflammatory response to bacterial invasion. This inflammatory response coincides with acute illness, and often in a measurable deviation of vital signs, such as a raised temperature and decreased blood pressure. In daily clinical practice, severity of the acute illness is assessed on basis of history, comorbidity and on severity of local and vital signs. It has become clear that severity of illness can also be more objectively expressed in biomarkers, which individually are associated with complicated course of disease, such as bacteraemia, need for ICU admission, time to defervescence and length of hospital stay. Although PCT and proADM were correlated to these clinical outcome parameters in our patients, they did not help predict outcome of the otherwise standardized antibiotic treatment, irrespective of a treatment duration of 7 or 14 days. The most likely reason is that all patients were treated for at least 7 days after which treatment success was high already (i.e., 89%). Thus, the lack of predictive value for treatment guidance is likely explained by the fact that historically, empiric treatment duration is based on the anticipated time to clinical recovery, while taking into account the inter-individual variety in severity of acute illness at the start of treatment (i.e. practically, adding some days of treatment to average recovery time). Thus, in our patients, the median time to defervescence was 2.0 (IQR 1–3) days while randomization for short or prolonged treatment duration did not start until day 7. In other words, one could have predicted that our biomarker approach might have been successful in guiding treatments up to one or a few days after clinical recovery, but it lacked the ability to do so after the minimum 7 days of treatment, by which a strong margin surpasses the time for the biomarker signature to return to normal. Differences between patients in severity of illness at presentation and corresponding biomarker levels are likely to have normalized after 7 days of treatment, and definitely after 14 days. It should be noted, that antimicrobial resistance played no role in our study; patients with a ciprofloxacin resistant uropathogen were excluded from randomization and received effective therapy based on culture results.

In the original study we described that although 7 days of treatment was inferior to 14 days in male patients in terms of short term clinical cure, there was no difference in the requirement for antibiotic retreatment for UTI during longer follow up (90 days) in both women and men [1].

A retrospective analysis of a large database of male veterans also found that treatment duration longer than 7 days was not associated with a reduction of UTI recurrence [18]. In this study, UTI recurrence was independently associated with age and comorbidity. It is likely that a subgroup of patients can be treated with an even shorter course of antibiotics than 7 days. Hypothetically, within the current time frame of treatment, there is a moment when the patient has recovered from the acute illness and the biomarker levels have dropped below a certain cut-off, likely leaving room for further limitation of treatment duration. It stands to reason that the time to this recovery is defined by host related factors, and therefore differs between patient subgroups. Biomarkers have the potential to objectively identify the optimal moment for cessation of therapy, irrespective of patient characteristics. Unfortunately, as reasoned above, our study design did not allow for further discrimination.

The main strength of this trial is its pragmatic nature reflecting daily clinical practice. We randomized patients between the up until then standard treatment duration for fUTI of 14 days and half of that length (7 days), and included enough subjects to demonstrate non-inferiority. We enrolled consecutive patients with fUTI, irrespective of age, gender and underlying medical condition. Different biomarker concentrations were available at the day of presentation and after three days of treatment, each representing different aspects of the physiological condition of the acute illness.

Limitations of our study include the design in randomizing patients between 7 and 14 days, not allowing for analysis of biomarker value in shorter antibiotic treatment. This was unforeseen, because at the start of the trial 14 days of therapy were standard clinical practice, and reduction to 7 days was already a great improvement. Furthermore, our sample size may have been too small to exclude a type II error.

To our knowledge, only one study addressing biomarker-guided antibiotic therapy in community acquired urinary tract infection has been published [10]. In this study, Drozdov et al. randomized both patients with cystitis (*N* = 36) and febrile urinary tract infections (*N* = 84) between PCT-guided or standard treatment. Overall, antibiotic exposure was reduced whereas adverse outcomes (clinical recurrence and rehospitalization rate) were similar in both groups. In the subgroup analysis of patients with fUTI/pyelonephritis, PCT-guided duration of antibiotic therapy was significantly shorter than standard care (7.5 vs. 11.0 days). In bacteraemic patients, recurrence rate (56% vs. 16%) and persistent infection after treatment (13% vs. 6%) were higher in the PCT guided group, although the distribution of patients with bacteraemia was not well balanced between the groups (45% vs. 21%) and numbers were too small to reach significance. Although this study by Drozdov et al. is limited by the small sample size and heterogenicity of patients, it supports the potential use of PCT-based approach in patients with community acquired fUTI to safely reduce antibiotic consumption.

Future interventional studies should be conducted to examine if PCT-guided duration of antibiotic treatment can indeed aid in distinguishing a subgroup of patients with fUTI who can be treated with antibiotics for even shorter than 7 days, i.e. those with rapid normalization of elevated biomarkers, in order to further decrease antibiotic exposure and limit development of antimicrobial resistance.

In conclusion, although the biomarkers PCT and MR-proADM are correlated to clinical parameters indicating disease severity, they did not predict treatment outcome in patients with community acquired febrile urinary tract infection, who are treated for 7 or 14 days with ciprofloxacin. In our secondary analysis, CRP had no added value in the management of fUTI.

## Abbreviations

AB: Antibiotics
AUC: Area under the curve
BP: Blood pressure
CI: Confidence interval
CRP: C-reactive protein
ED: Emergency department
fUTI: Febrile urinary tract infection
ICU: Intensive care unit
IQR: Interquartile range
MR-proADM: mid-regional proadrenomedullin
PCT: Procalcitonin
ROC: Receiver operating characteristics curves
SD: Standard deviation
TMP/SMX: Trimethoprim-sulfamethoxazole
TRACE: Time Resolved Amplified Cryptate Emission
